# Imitated Prosodic Fluency Predicts Reading Comprehension Ability in Good and Poor High School Readers

**DOI:** 10.3389/fpsyg.2016.01026

**Published:** 2016-07-19

**Authors:** Mara Breen, Lianne Kaswer, Julie A. Van Dyke, Jelena Krivokapić, Nicole Landi

**Affiliations:** ^1^Department of Psychology and Education, Mount Holyoke CollegeSouth Hadley, MA, USA; ^2^Haskins LaboratoriesNew Haven, CT, USA; ^3^Department of Linguistics, University of MichiganAnn Arbor MI, USA; ^4^Department of Psychological Sciences, University of ConnecticutStorrs, CT, USA

**Keywords:** prosody, reading comprehension, prosodic fluency, reading development, prosodic phrasing

## Abstract

Researchers have established a relationship between beginning readers' silent comprehension ability and their prosodic fluency, such that readers who read aloud with appropriate prosody tend to have higher scores on silent reading comprehension assessments. The current study was designed to investigate this relationship in two groups of high school readers: Specifically Poor Comprehenders (SPCs), who have adequate word level and phonological skills but poor reading comprehension ability, and a group of age- and decoding skill-matched controls. We compared the prosodic fluency of the two groups by determining how effectively they produced prosodic cues to syntactic and semantic structure in imitations of a model speaker's production of syntactically and semantically varied sentences. Analyses of pitch and duration patterns revealed that speakers in both groups produced the expected prosodic patterns; however, controls provided stronger durational cues to syntactic structure. These results demonstrate that the relationship between prosodic fluency and reading comprehension continues past the stage of early reading instruction. Moreover, they suggest that prosodically fluent speakers may also generate more fluent implicit prosodic representations during silent reading, leading to more effective comprehension.

## Introduction

Successful reading comprehension is a complex skill supported by a variety of subskills. Classic models of reading, such as Gough and Tunmer's (1986) Simple View of Reading, have argued that reading comprehension should be understood through its two main components: word recognition and general language ability. However, more recent research has highlighted the importance of specific components of reading comprehension, among them the role of reading fluency (e.g., Fuchs et al., 2001; Kuhn and Stahl, 2003; Schilling et al., 2007; Hudson et al., 2009). These studies and others suggest that readers who “sound good” when reading aloud are also good comprehenders.

A significant challenge to exploring the relationship between fluency and comprehension ability is in defining reading fluency. One definition of fluency has its roots in LaBerge and Samuels's (1974) automaticity theory of reading, which maintains that readers must have automated the lower-level processes of word recognition in order to devote cognitive resources to higher-order processes of comprehension. According to this view, reading fluency is defined as the number of words produced correctly in a specific time frame or for a specific passage (e.g., Fuchs et al., 2001; Torgesen et al., 2001; Jenkins et al., 2003; Daane et al., 2005; Torgesen and Hudson, 2006; Hudson et al., 2012). For example, in the widely used Dynamic Indicators of Basic Early Literacy Skills (DIBELS) assessment (Good and Kaminski, 2002), reading fluency is operationalized as the number of words read aloud accurately per minute in a standard passage.

Although fluency defined according to rate and accuracy measures has been shown to predict comprehension ability (Fuchs et al., 2001; Schilling et al., 2007), this definition fails to capture the importance of prosody to the perception of fluency in reading. Prosody, which describes variation in intonation, duration, rhythm, and intensity, is a critical component of perceived fluency in spoken language, as prosodic variation signals not only syntactic and semantic structure of sentences (e.g., Wagner and Watson, 2010), but also emotion (e.g., Cole, 2015). For example, Kuhn et al. (2010) note that, in addition to the role of rate and accuracy, prosodic fluency requires “appropriate expression or intonation coupled with phrasing that allows for the maintenance of meaning” (p. 233). Assessing this type of fluency is more difficult than assessing fluency according to rate and accuracy, however, as there are no simple methods for quantifying prosody. That is, although prosodic features like phrasing (i.e., cues to disjuncture) and stress (i.e., cues to prominence) are signaled through acoustic features like pitch, intensity, and duration, the relationship between the prosodic features and the acoustic measures is complex and variable (e.g., Wagner and Watson, 2010). Prosody researchers often deal with this complexity by relying on trained human annotators' perception of prosodic features, as in the Tones and Break Indices (ToBI) (Beckman and Ayers-Elam, 1997) and RaP (Rhythm and Pitch; Dilley and Brown, 2005) systems. However, the implementation of these systems is complex and time-consuming and requires considerable training to achieve acceptable inter-rater reliability (Breen et al., 2012).

Given the difficulty of measuring prosody directly, researchers have taken two main approaches to assessing prosodic fluency in readers' productions: (1) classifying utterances via impressionistic rating scales and (2) measuring acoustic features in specific sentence contexts. In the former method, a trained rater provides each reader with a score on a rating scale. Two such scales are the National Assessment of Educational Progress (NAEP) Oral Reading Fluency Scale (Pinnell et al., 1995), and the Multidimensional Fluency Scale (MFS) (Zutell and Rasinski, 1991). The NAEP Oral Reading Fluency Scale has four levels: The lowest level describes readers who primarily read word-by-word, while the highest level is assigned to readers who primarily read in “larger, meaningful phrase groups” that preserve the author's syntax. The MFS consists of four sub-sections for (i) expression and volume; (ii) phrasing; (iii) smoothness; and (iv) pace, and readers are given a score between one and four on each scale. Results from studies using both scales have demonstrated a positive correlation between reading comprehension and fluency. For example, Pinnell et al. (1995) reported that 4th graders who were rated higher on the Oral Reading Fluency Scale also scored higher on standardized tests of reading comprehension. Similarly, Rasinski et al. (2009) found a relationship between reading comprehension and fluency using a three-factor version of the MFS, such that more fluent readers in grades three, five, and seven also scored higher on standardized tests of reading comprehension.

Although rating scales such as these are valuable for their ease of implementation, and can provide educators with reliable indicators of student progress, there are challenges with their use. For example, any such scale will require considerable training for effective implementation (Rasinski et al., 2009) and require that raters exhibit acceptable levels of inter-rater agreement. More important, however, is that fact that subjective rating scales offer only a coarse measure of fluency, providing little sense of the specific aspects of prosody that contribute to effective comprehension. For example, the highest level of the Phrasing and Expression sub-scale of the MSF is characterized as follows:

“Reads with good expression and enthusiasm throughout the text. Sounds like natural language throughout the text. Varies expression and volume to match his or her interpretation. Generally well-phrased and meaningful; mostly in phrase, clause, and sentence units, with adequate attention to expression” (Rasinski et al., 2009, Figure 1).

**Figure 1:**
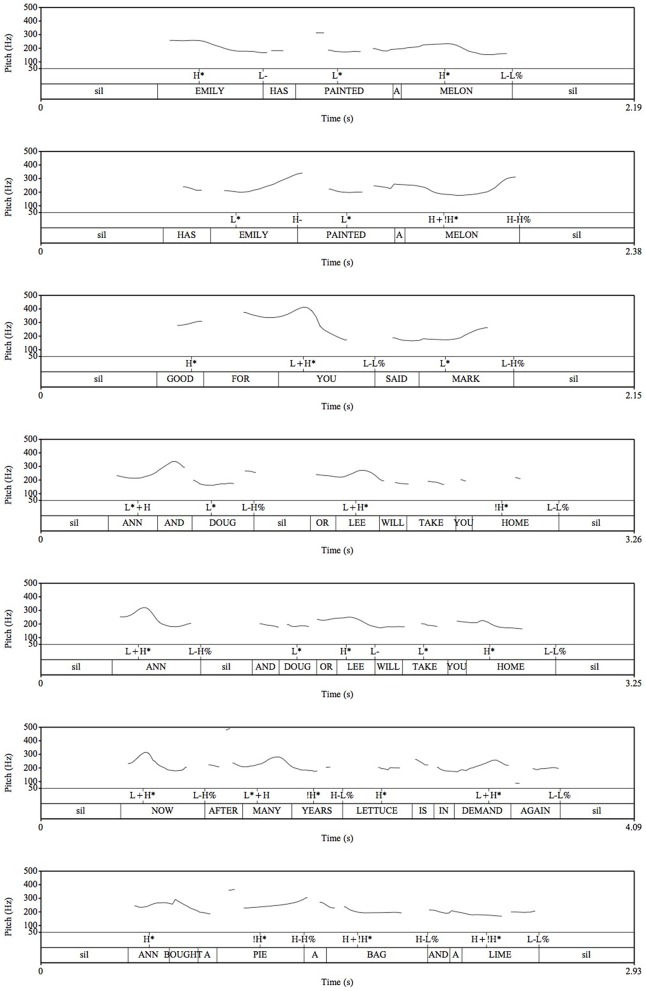
**Pitch tracks and Tones and Breaks (ToBI) annotations of one sentence from each stimulus type**. See text for description of labels.

This definition provides no information about the specific prosodic features that warrant a score at this level. For example, how are students realizing phrasing? And how are they varying their expression? Precise answers to these questions would move these assessments beyond the level of merely providing description to a position where actual pedagogical goals could be established. For example, specific skills, like the effective use of pitch contours, or appropriate phrasing cues, could be emphasized for those readers who lacked them. Thus, a chief goal of the current study is to understand how the prosodic productions of poor comprehenders compare to those of good comprehenders.

Fortunately, considerable work in recent years from the psycholinguistic literature (reviewed in Shattuck-Hufnagel and Turk, 1996; Cutler et al., 1997; Wagner and Watson, 2010) has assessed the relationship between prosody and acoustics, meaning we can now make specific predictions about how prosodically fluent readers will cue syntactic structure and semantic structure. For example, speakers often cue syntactic phrase boundaries through the employment of intonational phrase boundaries, signaled with a combination of duration and pitch cues, including lengthening of pre-boundary words (Lehiste et al., 1976; Selkirk, 1984; Price et al., 1991; Wightman et al., 1992; Ferreira, 1993; Schafer et al., 2000; Snedeker and Trueswell, 2003; Breen et al., 2010b), the presence of silence between words (Lehiste, 1973; Klatt, 1975; Cooper and Paccia-Cooper, 1980), and occasionally a pitch excursion, which can be either rising or falling, depending on the context (Streeter, 1978; Pierrehumbert, 1980): Declarative sentences end often end with a pitch fall, while interrogatives are signaled by a pitch rise (O'Shaughnessy, 1979; Chafe, 1988).

Extensive empirical investigation of the acoustic correlates of prosodic prominences, or accents, indicates that speakers routinely signal new or important discourse information with a combination of acoustic cues, including increased duration (Fry, 1955; Beckman, 1986; Breen et al., 2010a), increased intensity (Beckman, 1986; Turk and Sawusch, 1996; Kochanski et al., 2005; Breen et al., 2010a) and higher pitch (Lieberman, 1960; Cooper et al., 1985; Eady and Cooper, 1986; Breen et al., 2010a).

Using this knowledge about the relationship between phonological constructs like intonational phrase boundaries and prominences with acoustic cues like duration and pitch, some recent studies have explored prosodic fluency during reading using acoustic features measured with spectrographic analysis tools. Schwanenflugel et al. (2004) reported a large-scale study of 120 s- and third-graders (7–10-yr-olds) in which they objectively measured both pausing and pitch variables and assessed how those variables related to both word decoding and comprehension. Specifically, they measured (a) intersentential pause length means, (b) intersentential pause length variances, (c) intrasentential pause length means, (d) child-adult F0 sentence profile, and (e) sentence-final F0 declination. Their results demonstrated no significant relationship between fluency and overall reading comprehension, but they did find a relationship between prosodic fluency and word decoding, such that readers with poor decoding skills produced weaker pausing and pitch cues. This relationship makes sense, in that readers who have difficulty identifying words will likely not be able to devote the additional attentional resources to understanding the syntactic and semantic structure, and, therefore, their prosody will not signal these features. In order to ensure that differences in prosody production in the current study were not due to low-level word recognition, we assessed prosodic production across groups will holding decoding skill constant. Further, we employ a prosody imitation task to explore the direct relationship between prosodic production and comprehension without requiring word decoding.

Miller and Schwanenflugel (2006) further investigated the relationship between acoustic-prosodic features and reading comprehension ability in third-grade students using more syntactically complex items than those used by Schwanenflugel et al. (2004): (a) Basic declarative sentences, (b) Basic quotatives, (c) Wh-questions, (d) Yes-No questions, (e) Complex adjectival phrase commas, and (f) Phrase-final commas. Overall, they found that reading skill correlated with both phrasing and pitch behavior: Better readers made shorter pauses after sentence-final words and phrase-final commas than poor readers, and better readers produced larger pitch falls at the end of declarative sentences, as well as larger rises at the end of yes-no questions.

Further work by Schwanenflugel and colleagues replicated and extended the findings of a relationship between prosodic fluency and reading comprehension, demonstrating that children who exhibited good prosody (characterized by fewer inappropriate pauses) in first grade produced more adult-like prosody in second grade, and that adult-like prosody predicted later reading fluency scores (Miller and Schwanenflugel, 2008). Moreover, Schwanenflugel et al. (2015) demonstrated that children who exhibited better reading fluency (operationalized as faster and more accurate word recognition) produced stronger pitch cues to semantic and syntactic structure in sentences containing statements vs. questions, contrastive focus, and quotations. Specifically, good readers produced contrastive focus, direct quotes, and exclamations with higher pitch and greater intensity than poor readers, and produced larger (rising and falling) pitch excursions for statements and yes-no questions, respectively, than poor readers. Finally, Benjamin et al. (2013) demonstrated significant a strong relationship between fluency and comprehension using the Comprehensive Oral Fluency Scale (CORFS), a rating scale empirically derived from spectrographic measures of prosodic fluency.

Results from Schwanenflugel and colleagues demonstrate that prosodic fluency is predictive of reading comprehension ability for beginning readers between the ages of 6 and 9 (grades 1 through 3). However, the relationship between older children's prosodic fluency and comprehension skills has been the subject of little research, in part because of an assumption that, by secondary school, readers have reached an acceptable level of proficiency (e.g., Chall, 1983). But there is reason to suspect that the relationship between prosodic fluency and comprehension ability continues to change as students' proficiency improves, and as their prosodic skills improve (Kuhn et al., 2010). This work is especially important given the finding that prosodic fluency is a more effective predictor of reading comprehension for difficult constructions than simpler ones (Benjamin and Schwanenflugel, 2010), suggesting that prosodic fluency deficits will be even more challenging for older readers, who are routinely exposed to more syntactically and semantically complex texts than younger readers.

Studies investigating the relationship between prosodic fluency and reading comprehension in older children have largely utilized ratings scales. Karlin (1985) used an impressionistic rating scale to assess pitch, stress, and phrasing behavior in a set of college students, and found no consistent relationship between prosodic fluency and reading comprehension ability. However, Rasinski et al. (2005) found that 9th graders' fluency (operationalized as the number of words read out loud correctly in a minute) was positively correlated with the same students' scores on a standardized silent reading test. In follow-up work, Paige et al. (2014) demonstrated that 9th graders' silent reading comprehension was correlated with prosodic fluency as measured by the MFS.

Although we are not aware of any prior studies investigating the relationship between high school readers' comprehension and prosodic fluency using the types of acoustic measures reported by Schwanenflugel and colleagues, Binder et al. (2013) presented evidence that this relationship holds for adult readers (college-aged and above). Replicating results from studies on children, low-skilled adult readers (as measured by phonemic segmentation knowledge, non-word reading accuracy, word identification accuracy, and passage comprehension) produced more inappropriate pauses and smaller pitch variability across questions than skilled adult readers.

The studies described above demonstrate a positive correlational relationship between prosodic fluency and reading comprehension in learning readers, experienced (high school) readers, and adults. However, there are important remaining questions from these studies. First and foremost, differences between good and poor comprehenders observed in previous studies may be due to differences in word decoding skill. For example, poor decoders (both children and adults) have been shown to pause inappropriately more than good decoders, and to exhibit less pitch variation (Miller and Schwanenflugel, 2008; Binder et al., 2013; Schwanenflugel et al., 2015). These effects may not be due to comprehension *per se*, but rather to lower-level word identification problems for less-skilled readers.

In order to objectively assess the relationship between prosodic fluency and comprehension, without confounding fluency with decoding difficulty, the current study differed in two significant ways from previous studies. First, we compared the prosody of high school readers who were good and poor comprehenders, matched on word decoding ability (all with standard scores indicating *at least* average ability). The latter group, which has been referred to as Specifically Poor Comprehenders (SPCs)^1^ or simply Poor Comprehenders (Nation and Snowling, 1998; Landi, 2010), having intact decoding skills, but selective deficits in reading and listening comprehension. This group is of particular interest because they have identified weaknesses in a number of comprehension subskills such as vocabulary and grammatical processing, however, significant gaps remain in our understanding of factors that contribute to these weaknesses (Landi and Ryherd, submitted). Second, compared to prior studies which have utilized spontaneous productions of sentence material, we utilized an imitation paradigm in which participants repeated sentences which had been produced by a model speaker. In this way, we can assess the extent to which participants produce prosodic cues to syntactic and semantic structure without the added challenge of identifying the words. Because our participants have an acoustic model of the words (as well as the ability to read them off the screen), any differences in prosody between good and poor comprehenders cannot be due to word recognition differences.

An advantage of investigating the relationship between prosodic fluency and reading comprehension in older readers is that they tend to realize more features of adult prosody than younger readers (Cruttenden, 1985; Katz et al., 1996; Wells et al., 2004; Patel and Grigos, 2006). Therefore, we can use their productions to investigate more subtle relationships between prosodic fluency and comprehension ability. For example, Schwanenflugel et al. (2004) measured intersentential and intrasentential pause length, with the prediction that, in general, good readers will produce short pauses within sentences and longer pauses at the ends of sentences. This gross measure may have effectively captured variation between beginning readers' fluency levels, but it fails to capture the true relationship between syntactic structure and phrasing. Using older readers, we predicted that good comprehenders would show sensitivity to more complex phrasing relationships, and would use prosody to signal syntactic structure more effectively that SPCs.

Imitation tasks have proven to be effective in eliciting successful mimicry for phonetic variables (Goldinger, 1998; German, 2012; German et al., 2013). Participants have also successfully imitated the phonology of model sentences (Braun et al., 2006; Cole and Shattuck-Hufnagel, 2011). Cole and Shattuck-Hufnagel (2011) measured both the phonological structure and the phonetic variables of their participants' imitations and found that although the overall prosodic structure (e.g., the location of phrase boundaries) generally remained intact, certain non-intonation phonetic cues such as pause duration were imitated with different levels of reliability, such that boundaries were more reliably imitated than accents. Other research has shown that phonetic cues are more likely to be imitated if they are relevant to the phonology of the sentence (Nye and Fowler, 2003; Mitterer and Ernestus, 2008). These results suggest that, when imitating, speakers will reproduce the global prosodic contour of the speaker they are imitating, but may also produce individual variation in their faithfulness to the specific acoustic realization of that contour.

In the current study, SPCs and decoding skill-matched Controls were recorded reproducing a model speaker's production of target sentences containing a variety of syntactic structures based largely on the materials employed by Miller and Schwanenflugel (2006) (**Table 2**). If readers' comprehension ability is related to their prosodic fluency, we predicted that SPCs, who have a selective reading comprehension deficit, would not realize these features as effectively as Controls.

## Methods

### Participants

Thirty-two participants contributed data to the analyses below. All were high school students between the ages of 13 and 19, recruited through local advertisements and flyers distributed throughout New Haven and Fairfield counties in Connecticut. All procedures were approved by the Yale University Human Investigations Committee. Only those students who gave their assent to participate and whose guardians consented to testing were admitted to the study. Participants were compensated $25 per hour for their participation. No participants had been diagnosed with learning or reading disabilities, and all were native speakers of American English who had not been exposed to another language before the age of seven. A total of 49 participants completed the experiment, and from that larger set we selected a group of SPCs (*N* = 16) and a group of control participants (*N* = 16) who met our criteria, as described below.

Participants in the SPC group had standardized scores of reading comprehension below 90, as measured by the Kaufman Test of Educational Achievement (KTEA; Kaufman and Kaufman, 2004); participants in the control group had comprehension scores of 100 or above (Table 1). The two groups were matched on age, gender composition, and phonetic decoding ability [as measured by the Word Attack subtest (WA) of the Woodcock Johnson III] (Woodcock et al., 2001), but differed significantly in their reading comprehension scores. Most participants had performance IQ at or above the normal range (85–115), as measured by the Weschler Abbreviated Scale of Intelligence II (WASI; Psychological Corporation, 1999)^2^. However, we include one SPC with slightly below average performance IQ (see Table 1). In general it has been found that SPCs are more likely to score near the low-average end of the distribution or slightly below average (e.g., Nation et al., 2002), though there is no direct relationship between IQ and language function for children who score within the range reported here^3^. Moreover, we tested whether IQ improved model fit in all of the statistical models described below, but it never did.

**Table 1:** **Descriptive Statistics of SPCs and Controls, with means and standard deviations in parenthesis**.

	**Control Group**	**SPCs**	**Significance test**
N	16	16	
Age	16.6 (1.8)	17.3 (1.4)	^*t*^(28.8) = −1.2, *p* = 0.26
Word attack standard score	109 (6.0)	109 (6.5)	^*t*^(29.8) = −0.1, *p* = 0.96
IQ (WASI)	109 (15.6)	93.8 (6.0)	^*t*^(19.3) = 3.4, *p* < 0.01
KTEA standard score	110 (8.1)	84.3 (4.9)	^*t*^(24.6) = 10.4, *p* < 0.001
Number of Female	9	9	

### Materials

In order to test the hypothesis that prosodic fluency is related to reading comprehension ability, we constructed a set of seven sentence types, which varied in their syntactic and semantic structure. Each sentence type was designed to elicit the production of a variety of phrasing and intonation patterns. An example of each type of construction appears in Table 2. The sentences were of seven different types: (a) declarative statements, (b) yes-no questions, (c) basic quotatives, (d) ambiguous coordinate structures, (e) relative clauses, and (f) unambiguous coordinate structures. The stimuli were presented to the participants with normal punctuation. The full list of experimental stimuli can be found in Appendix in Supplementary Materials.

**Table 2:** **Examples of sentence stimuli and associated acoustic features of interest**.

	**Construction type**	**Example**	**Acoustic features of interest**
1	Declarative statement	Emily has painted a melon.	Pitch
2	Yes/No question	Has Emily painted a melon?	Pitch
3	Basic quotative	“That sounds wonderful!” said Jane.	Pitch
4	Ambiguous coordinate structure	Ann(,) and Bobby(,) or Nancy, will come.	Duration
5	Relative clause	The room, which had a red chair, caught Mandy's eye.	Pitch and duration
6	Unambiguous coordinate structure	Ann has a dog, a pen, and a mug.	Pitch

#### Declarative statements

The sentences in this set minimally included a subject, verb, and direct object (e.g., Emily has painted a melon). In addition, 2 of the 10 included a sentence-final adverbial phrase (e.g., on Monday).

Previous research demonstrates that these structures tend to be produced with a falling pitch across the sentence (Lieberman, 1967; Cruttenden, 1981; Eady and Cooper, 1986; Hirst and Di Cristo, 1998). Moreover, the size of the pitch declination has been shown to correlate with reading comprehension skill such that better comprehenders produce larger falls at the end of declarative sentences. (Dowhower, 1987; Schwanenflugel et al., 2004). Therefore, we predict that SPCs will produce smaller pitch declination for declarative sentences than controls.

#### Yes-no questions

The sentences in this set were derived from the declarative sentences above. For example, “Emily has painted a melon” became “Has Emily painted a melon?” Yes-no questions such as these have been shown to elicit a sentence-final pitch rise in adults (O'Shaughnessy, 1979; Chafe, 1988). Moreover, the size of the rise has been shown to be positively correlated with reading comprehension ability, such that elementary school readers with better comprehension scores produced larger final rises (Miller and Schwanenflugel, 2006). Therefore, we predict that SPCs will produce smaller pitch rises for yes-no questions than controls.

#### Basic quotatives

The sentences in this set included simple instances of directly reported speech, using basic vocalization verbs such as “said,” “responded,” “replied.” The directly reported speech was always first in the sentence, followed by the attributive phrase (e.g., “That sounds wonderful,” said Jane). Prior work on adult prosody has demonstrated that, in sentences like these, directly reported speech is produced with more pitch variation than the attributive phrase (Jansen et al., 2001). Therefore, we predict that SPCs will produce a smaller difference in pitch variability between the reported speech and attributive speech than those in the Control group.

#### Ambiguous coordinate structure

The sentences in this set included globally ambiguous sentences with coordinate structures that can be disambiguated prosodically (e.g., Ann and Bobby or Nancy will come). Every sentence included a list of three subjects separated by the conjunctions “and” and “or” in the same order. The model speaker produced two versions of each of these sentences: In the *Two-One* phrasing condition, she produced a phrase boundary after Bobby, as in (1), indicating that both “Ann and Bobby” will come or only “Nancy” will come. In the *Two-Two* phrasing condition, she produced a phrase boundary after “Ann,” as in (2), indicating that either “Ann and Bobby” will come or “Ann and Nancy” will come (Wagner, 2005). One of the primary acoustic correlates of a phrase boundary is an increased duration of the pre-boundary word (e.g., Wightman et al., 1992). We predict that the duration of nouns preceding boundaries in (1, 2) will be longer than nouns preceding non-boundaries, such that “Ann” will be longer in two-two disambiguation than in two-one disambiguation, whereas “Bobby” will be longer in two-one disambiguation than two-two disambiguation. Furthermore, we predict that these patterns will be modulated by reading comprehension ability. Specifically, we predict that SPCs will produce weaker duration cues to boundaries than controls.

(1) Ann and Bobby || or Nancy will come
(2) Ann || and Bobby or Nancy will come

#### Relative clause

This set included syntactically complex sentences containing non-restrictive relative clauses^4^ (e.g., The room, which had a red chair, caught Mandy's eye). Previous work demonstrates that parentheticals are often produced in separate phrases from the rest of the sentence (Dehé, 2007). Adult readers tend to mark the end of a parenthetical clause with a pitch shift, and with a longer pause than the boundary preceding the clause (Kutik et al., 1983). Moreover, Watson and Gibson (2004) observed that speakers are more likely to produce a boundary preceding a non-restrictive relative clause than a restrictive relative clause. We predict that all participants will produce similar cues to syntactic phrasing, but that controls will produce greater differences in duration and pitch between boundary and non-boundary words than SPCs.

#### Unambiguous coordinate structure

The sentences in this set always included three nouns (e.g., Emily has a dog, a pen, and a mug). Miller and Schwanenflugel (2006) tested similar sentences which included a series of three adjectives (e.g., Frog and Toad were happy, playful, curious animal friends) and demonstrated that more skilled readers produced larger pitch variation within the adjectives in the series as compared to the preceding context. We therefore measured pitch variation in the conjuncts as compared to the non-conjuncts. We predict that the SPCs will produce a smaller difference between the average pitch variation in the conjuncts (a dog and a pen) as compared to non-conjuncts (Emily and has), while the controls will produce a comparatively larger difference.

### Procedure

Before beginning the experiment, participants completed questionnaires on education, family, medical, and language history. In addition, they were administered a sequence of standardized assessments: Word decoding ability was assessed with the Word Attack subtest of the Woodcock Johnson Tests of Achievement (Woodcock et al., 2001); Reading comprehension was measured using the standard score of the Kaufman Test of Educational Achievement (Kaufman and Kaufman, 2004); Finally, IQ was measured by the Wechsler Abbreviated Scale of Intelligence (Wechsler, 1999). Administration of these assessments took approximately 40 min.

After assessment, participants were seated in front of a computer monitor and were fitted with headphones. Responses were recorded at a sampling rate above 44,100 Hz using a Sennheiser ME66 cardioid microphone positioned beside the monitor, aimed in the direction of the participant's head. Stimuli were presented electronically using the E-Prime 2.0 software (Psychology Software Tools^5^, Pittsburgh, PA).

Participants were then introduced to the prosody repetition paradigm, and instructed that they should produce the sentences they heard with a particular focus on replicating the prosodic structure. To make their task clear, participants heard an example trial where a male voice produced the sentence “Mary came home” with the typical prosody of a declarative statement, i.e., with a fall in fundamental frequency (F0) on “home” (Lieberman, 1967; Cruttenden, 1981; Eady and Cooper, 1986; Hirst and Di Cristo, 1998). The participants then heard examples of an appropriate repetition and an inappropriate repetition, produced by a female speaker: the first was again produced with a final F0 fall; the second was produced with a F0 rise on “home.”

After participants indicated their understanding of the procedure, they began the experiment. On each trial, participants were instructed to listen to a female speaker's voice over the headphones and repeat the sentence aloud into the microphone, with emphasis on imitating the presented prosodic structure. The sentence was displayed on the screen during the audio presentation, so that participants would not need to hold all of the words in memory. Participants were permitted to repeat the playback of the model speaker's production once on each trial, for a total of two presentations. Following the first (or second) iteration, participants pressed a key to begin recording and spoke the sentence aloud. When finished, the participant pressed a key to stop the recording and pressed the same key again to continue on to the next sentence. They received no feedback on their performance.

Participants completed three blocks with 86 stimulus sentences (see Appendix in Supplementary Materials) randomly presented in each block, for a total of 258 sentences each. Each block consisted of the same set of 86 sentences with the same prosodic structures presented, randomized within each block. There were two breaks provided in between each block of the experiment. The participants were encouraged to take a break, but could refuse. The entire session lasted ~45 min.

## Results

The three versions of each of 258 sentences produced by 32 speakers resulted in a possible total of 8256 productions. Our analyses were conducted on 8203. Fifty-three files (52 from one participant; one from another) were missing because the productions were unidentifiable. We used the Prosodylab-Aligner (Gorman et al., 2011) to force align the words from the target sentences with their waveforms. Using Praat (Boersma and Weenink, 2011), we extracted acoustic features from each word in each sentence. These pitch features were minimum F0, maximum F0, and mean F0 values at 10 equal-spaced intervals across the word. Word duration was defined as the duration of the word itself and any following silence.

In order to ensure that the model speaker was producing the intended prosody for each structure and to corroborate the acoustic features reported below, we enlisted a ToBI expert to generate ToBI annotations of one sentence from each of the sentence types (Figure 1). In every case, the model speaker produced the sentences with the predicted acoustic contours: She produced the final word in the Declarative sentence with a pitch fall (L-L%), and the final word in the Yes-No question with a rise (H-H%); she produced a larger pitch excursion for the quote portion of the Quotative sentences than the attributive phrase; in the Ambiguous Coordinate Structure sentences, she produced an intonational phrase (IP) boundary (as indicated by the presence of a H% boundary tone) after word one in the Two-One construction, and an IP boundary after word two in the Two-Two construction. In the relative clause constructions, the model speaker produced IP boundaries (4′s) at the beginning and end of the relative clause. Finally, in the unambiguous coordinate sentences, the model speaker produced IP boundaries after each conjunct.

In order to compare pitch values across speakers, we converted raw F0 values (measured in Hz) to semitones. In order to compare duration values across speakers, we normalized duration values with the following procedure: we subtracted a speaker-specific average from each value and divided the result by a speaker-specific standard deviation. We then excluded from analysis any normalized duration values which were three standard deviations or more above or below the mean.

In the analyses that follow, we present a series of statistical tests designed to determine whether speakers in our study implemented a particular prosodic pattern. In each case, we test a prosodic feature from the literature where researchers have previously identified a significant difference between the values of a specific acoustic feature on the same material across two contexts. Our models were designed to determine (a) whether our participants realized the acoustic difference in question and (b) whether the participants in the control group realized the difference more strongly than the SPCs.

To test our predictions, we modeled the data on a trial-by-trial basis with a series of mixed-effects linear regressions. In every model, the dependent variable was a continuous acoustic measure. The fixed effects in each model were the syntactic/semantic manipulation, the experimental group, and the interaction of these factors^6^. In addition, we included random effects of participant and item. We also investigated whether random slopes improved model fit using the following procedure: First, we attempted to fit a fully saturated model with random intercepts for subject and item as well as random slopes for both the main effects and the interaction. This fully-saturated model never converged, and so we iteratively removed terms from the random effects structure which accounted for the least variance in the non-converged model. In addition, once the model converged, we compared each model to a less complex model nested within it to determine whether the additional terms in the random effects structure were justified according to the procedure specified by Baayen (2008). The more complex model was only used if it significantly improved model fit. Condition means for the acoustic measures of interest from each structure type appear in Table 3. The parameters of the best fitting models appear in Table 4.

**Table 3:** **Means (and standard errors) of the acoustic measure of interest for each sentence type**.

**Syntactic structure: acoustic measure**		**Model speaker**	**Controls**	**SPCs**
Statement vs. Y-N Ques: Pitch	Declarative	6.42 (0.60)	1.37 (0.23)	1.18 (0.15)
	Yes-No question	−4.52 (1.63)	−3.39 (0.25)	−3.04 (0.19)
Basic quotative: pitch	Attributive phrase	6.27 (0.80)	5.17 (0.17)	4.71 (0.16)
	Quote	7.13 (1.1)	5.63 (0.18)	4.70 (0.16)
Ambiguous coordinate: duration “Ann”	Boundary	1.46 (0.22)	1.09 (0.05)	0.65 (0.05)
	No boundary	−0.39 (0.11)	−0.32 (0.02)	−0.30 (0.03)
Ambiguous coordinate: duration “Bobby”	Boundary	1.13 (0.23)	1.18 (0.04)	0.65 (0.04)
	No boundary	−0.16 (0.20)	−0.10 (0.03)	0.01 (0.03)
Relative clause: duration	Boundary	1.13 (0.24)	1.18 (0.04)	0.65 (0.04)
	No boundary	−0.16 (0.20)	−0.11 (0.03)	0.01 (0.03)
Relative clause: pitch	Boundary	8.75 (0.73)	7.34 (0.122)	5.89 (0.11)
	No boundary	3.42 (0.33)	3.67 (0.09)	3.20 (0.08)
Unambiguous coordinate: duration	Boundary	0.57 (0.09)	0.79 (0.03)	0.68 (0.03)
	No boundary	−0.40 (0.10)	−0.42 (0.02)	−0.33 (0.03)
Unambiguous coordinate: pitch	Boundary	7.21 (1.43)	6.10 (0.19)	5.28 (0.12)
	No boundary	4.17 (0.43)	4.00 (0.10)	3.72 (0.09)

**Table 4:** **Parameter estimates of regression models**.

**Syntactic contrast: acoustic feature**	**Est**.	**SE**	***t***	***p***
**STATEMENT VS. YES-NO Q: PITCH**				
Intercept	−0.78	0.49	−1.59	n.s.
Statement vs. Yes-No	−4.13	0.71	−5.83	^*^
Group	0.23	0.52	0.45	n.s.
Sentence type × Group	0.77	0.87	0.88	n.s.
**BASIC QUOTATIVE: PITCH**				
Intercept	4.94	0.23	21.32	^*^
Attributive (non-quote) vs. Quote	0.22	0.53	0.43	n.s.
Group (High and Low)	0.46	0.39	1.17	n.s.
Word category × Group	0.46	0.31	1.50	n.s.
**TWO-TWO AND TWO-ONE PHRASING: DURATION “ANN”**				
Intercept	0.20	0.10	1.92	n.s.
Boundary vs. No boundary	−1.00	0.19	−5.30	^*^
Group (High and Low)	0.21	0.08	2.54	^*^
Boundary × Group	−0.44	0.18	−2.42	^*^
**TWO-TWO AND TWO-ONE PHRASING: DURATION “BOBBY”**				
Intercept	0.40	0.03	13.76	^*^
Boundary vs. No boundary	0.13	0.02	6.73	^*^
Group (High and Low)	0.04	0.02	2.56	^*^
Boundary × Group	0.11	0.01	9.63	^*^
**RELATIVE CLAUSE: DURATION**				
Intercept	0.24	0.07	3.43	^*^
Boundary vs No Boundary	−1.78	0.13	−13.62	^*^
Group (High and Low)	−0.08	0.04	−1.87	^∧^
Boundary × Group	0.37	0.08	4.58	^*^
**RELATIVE CLAUSE: PITCH**				
Intercept	3.43	0.17	20.12	^*^
Boundary vs No Boundary	3.18	0.17	18.55	^*^
Group (High and Low)	0.47	0.22	2.13	^*^
Boundary × Group	0.98	0.34	2.87	^*^
**UNAMBIGUOUS COORDINATE: DURATION**				
Intercept	0.18	0.07	2.42	^*^
Boundary vs. No Boundary	−1.11	0.08	−13.46	^*^
Group (High and Low)	0.01	0.03	0.35	n.s.
Boundary × Group	−0.20	0.06	−3.31	^*^
**UNAMBIGUOUS COORDINATE: PITCH**				
Intercept	4.77	0.18	27.22	^*^
Boundary vs. No Boundary	−1.83	0.17	−10.47	^*^
Group (High and Low)	0.55	0.28	1.96	^∧^
Boundary × Group	−0.53	0.35	−1.52	n.s.

Random slopes were used in each model, so pMCMC cannot be calculated;

* indicates estimated significance beyond the 0.05 level;

∧ *indicates marginal significance; n.s. indicates a non-significant effect*.

### Statement vs. yes-no question: Pitch slope

In order to determine whether Controls and SPCs differed in the extent to which they realized pitch falls on declarative statements and pitch rises on yes-no questions, we compared the slope of the pitch on the final word of the statements and yes-no questions across groups. For each production, we computed the pitch slope of the final word by subtracting the raw F0 80% of the way into the word from the raw F0 measured 20% of the way into the word. We then transformed the raw Hz value into semitones, in order to make comparisons across speakers. In this way, a positive pitch slope corresponded to a pitch declination, while a negative pitch slope corresponded to a rising pitch.

**Figure 2:**
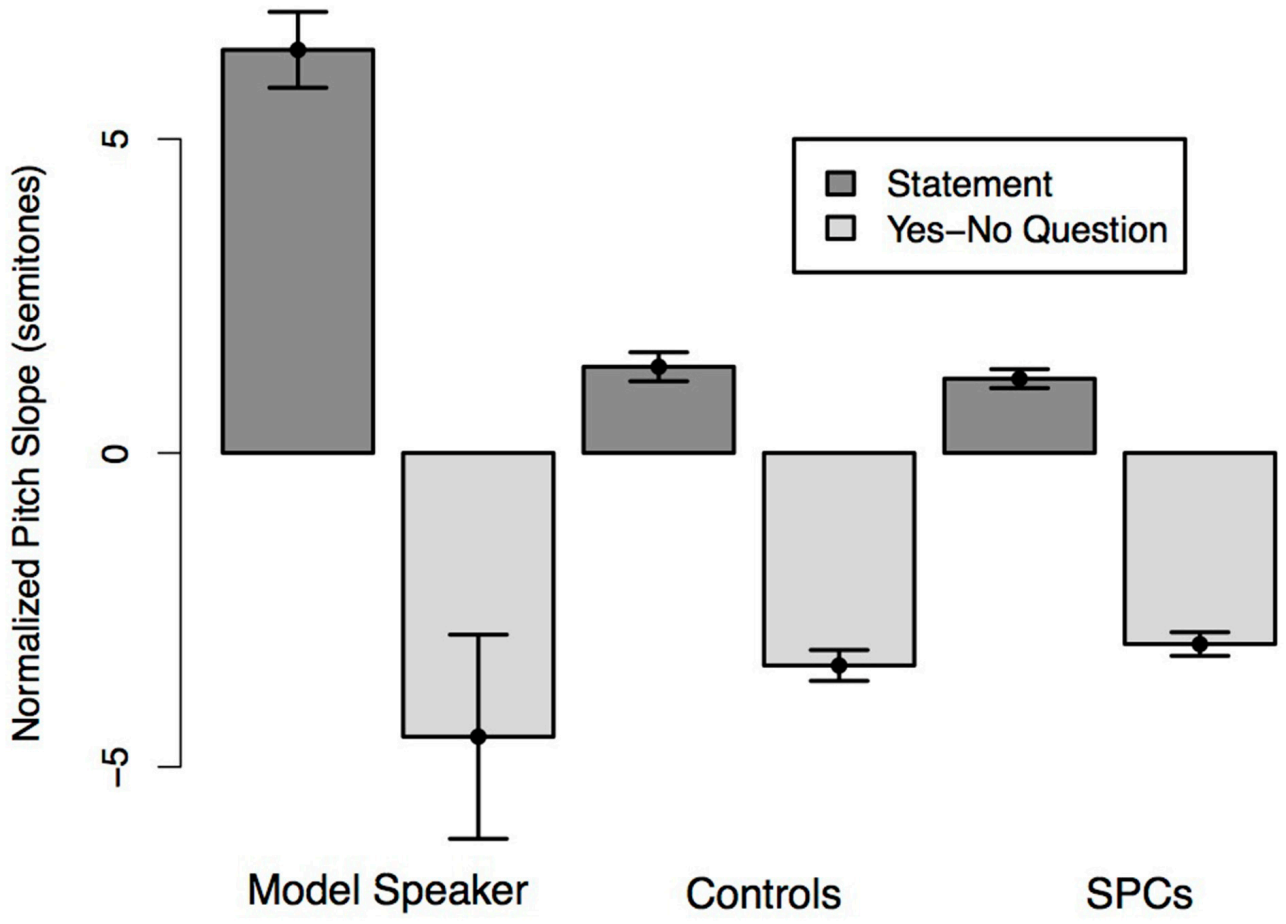
**Normalized pitch slope on the final word of Statements and Yes-No Questions as produced by the Model Speaker, Control Speakers, and SPCs**. A positive pitch slope corresponds to a declination in F0 over the word; a negative slope corresponds to a F0 rise. Error bars represent standard errors.

We predicted that all speakers would produce the final word of statements with a higher (i.e., more positive) pitch slope than the final word of Yes-No Questions and that this effect would be moderated by Group such that the controls would produce a larger difference in slope than SPCs. A mixed-effects linear regression predicting pitch slope from sentence type (Statement, Yes-No Question) and Group demonstrated a main effect of sentence type, such that the pitch slope was higher (i.e., positive) for statements, and lower (i.e., negative) for Yes-No questions (*t* = −5.83). There was no effect of Group on pitch slope (*t* = 0.45), indicating that the slopes didn't differ significantly across Controls and SPCs. Likewise, Sentence Type and Group did not interact (*t* = 0.88), meaning that the two groups did not differ in terms of how they realized a pitch change on the final word of the two sentences (Figure 2).

### Basic quotative: Pitch variability

In order to determine whether Controls and SPCs differed in the extent to which they realized pitch variability differently across direct quotes and attributive phrases, we compared pitch variability within the direct quote to that of the attributive phrase. For each production, we computed a value of pitch variability for the words within the quote (e.g., That, sounds, and wonderful) and the words in the attributive phrase (e.g., said and Jane). To do this, we subtracted the raw minimum F0 from the raw maximum F0 for each word in the sentence, transformed this value into semitones, and averaged this pitch variability value over the words within the quote and attributive phrase separately for each production from each speaker.

**Figure 3:**
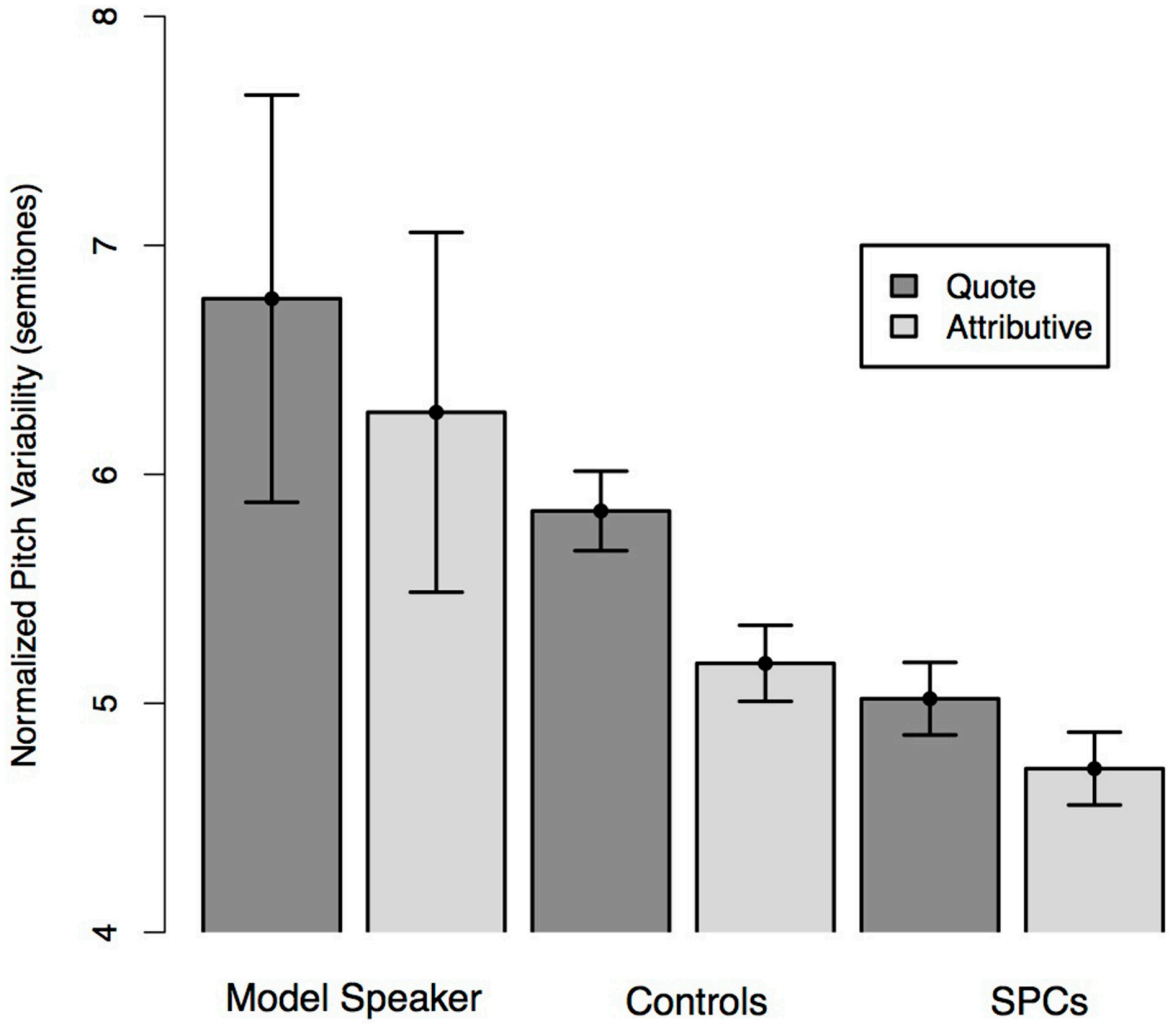
**Normalized pitch variability on the quote and attributive phrases of Simple Quotative sentences as produced by the Model Speaker, Control Speakers, and SPCs**. Error bars represent standard errors.

We predicted that all speakers would produce more pitch variability for the quote than the attributive phrase and that this effect would be moderated by Group such that the controls would produce a larger difference in variability than Poor Comprehenders. A mixed effects linear regression predicting pitch variability from phrase type (Quote, Attributive phrase) and Group demonstrated no main effect of phrase type (*t* = 0.43), such that, contrary to our prediction, participants did not produce the quotes with greater variability than the attributive phrases. There was no main effect of Group on pitch variability (*t* = 1.17), and no interaction between phrase type and Group (*t* = 1.50), though Controls produced a numerically larger difference in pitch variability for the quotes than the attributive phrases (Figure 3).

### Ambiguous coordinate structure: Duration

In order to determine whether Controls and SPCs differed in the extent to which they realized the phrasing of sentences with ambiguous coordinate structure, we compared differences in the duration of the same words depending on the intended syntactic structure. For each ambiguous sentence, we computed the difference between the normalized duration of the first and second conjunct depending on whether they were produced with phrasing that corresponded to the “Two-One” interpretation or the “Two-Two” interpretation. To do this, we carried out two analyses. In the first, we computed, for every sentence, the normalized duration (plus any following silence) of the first conjunct (e.g., Ann), which we predicted would be lengthened in the two-one condition compared to the two-two condition. In the second analysis, we computed, for every sentence, the normalized duration (plus any following silence) of the second conjunct (e.g., Bobby), which we predicted would be lengthened in the two-two condition compared to the two-one condition.

**Figure 4:**
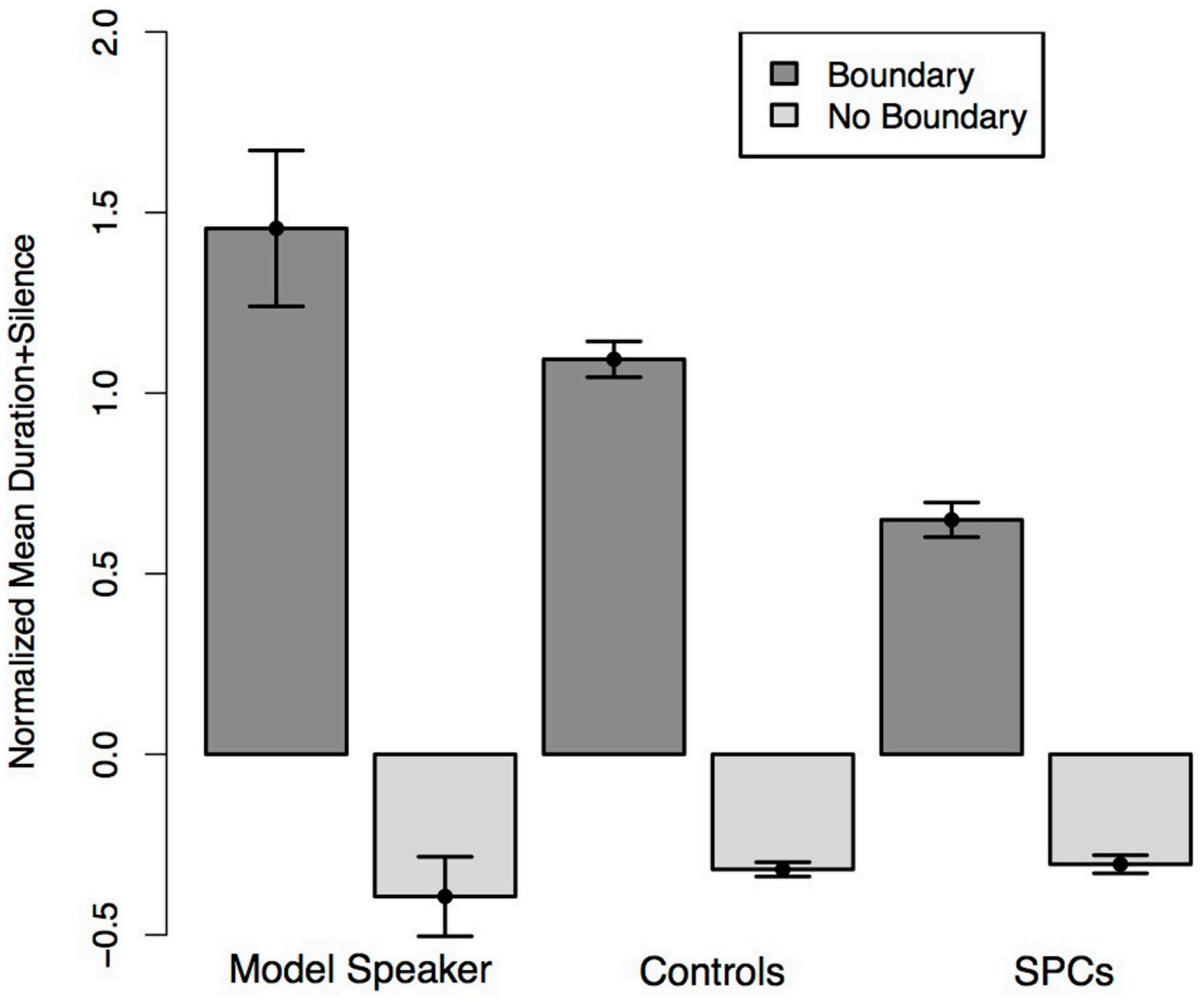
**Normalized average duration plus silence of the first conjunct of the Ambiguous Coordinate Structure sentences depending on whether the speaker intended the two-one (No Boundary) or two-two (Boundary) structure as produced by the Model Speaker, Controls, and SPCs**. Error bars represent standard errors.

We predicted that all speakers would reproduce the phrasing of the model speaker, such that the duration of the first conjunct would be longer for the two-one condition than the two-two condition, and the duration of the second conjunct would be longer in the two-two condition than the two-one condition. We also predicted that these effects would be moderated by Group such that Controls would produce larger duration differences across condition than SPCs. We conducted two mixed-effects linear regressions to test our hypotheses. In the first analysis, we predicted the duration of conjunct one (Ann) from condition (two-one, two-two) and Group. This analysis revealed a main effect of condition (*t* = −5.30), such that participants in both groups produced the first conjunct with longer duration in the two-two condition than the two-one condition. We also observed an effect of Group (*t* = 2.54) driven by the fact that members of the Control group produced all conjuncts, regardless of whether the coincided with a boundary, with longer durations than the SPCs. Finally, we observed an interaction between condition and Group (*t* = −2.42) indicating that controls produced larger differences between the duration of second conjunct across conditions than did the SPCs (Figure 4), thereby providing a stronger signal to the presence of an intonational boundary.

**Figure 5:**
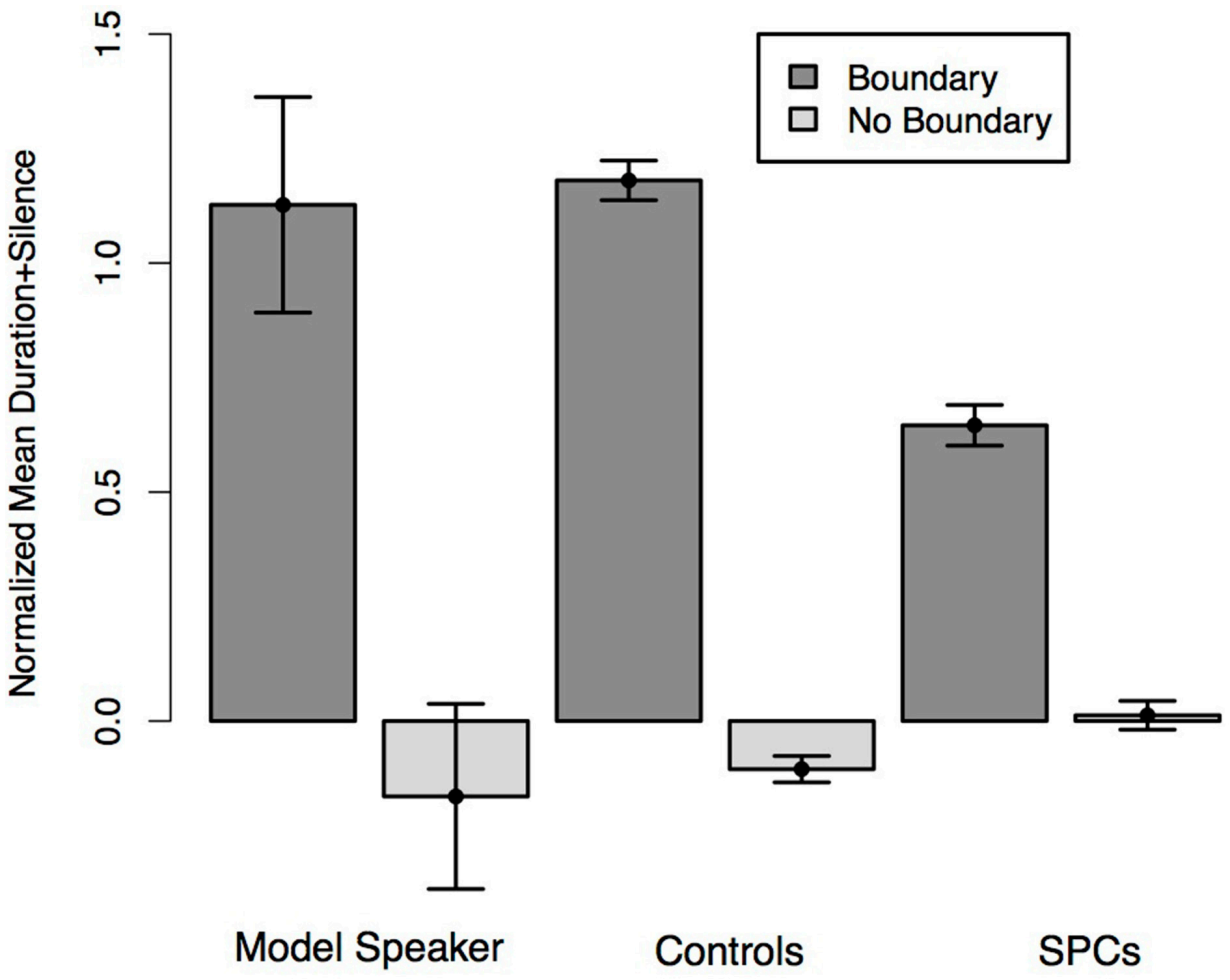
**Normalized average duration plus silence of the second conjunct of the Ambiguous Coordinate Structure sentences depending on whether the speaker intended the two-one (Boundary) or two-two (No Boundary) structure as produced by the Model Speaker, Controls, and SPCs**. Error bars represent standard errors.

In the second analysis, we predicted the duration of the second conjunct (Bobby) for condition (two-one, two-two) and Group. This analysis revealed a significant main effect of condition (*t* = 6.73), such that all speakers produced the second conjunct with longer duration in the two-one condition than the two-two condition, as well as a main effect of group (*t* = 2.56) such that Controls produced both the second conjunct in both conditions with longer durations than SPCs. Finally, there was a significant interaction between condition and Group (*t* = 9.63) such that Controls produced a larger duration difference across conditions than SPCs (Figure 5), again providing a stronger signal to the presence of an intonational boundary.

### Relative clause: Duration

In order to determine whether Controls and SPCs differed in the extent to which they produced durational cues to phrase boundaries, we compared the duration of words that occurred at a hypothesized boundary location to those that did not. For each production, we computed one value for the average normalized duration of words that occurred at a boundary location (e.g., room, chair) and a second value for the average normalized duration of all words that occurred at a non-boundary location (e.g., The, which, red).

**Figure 6:**
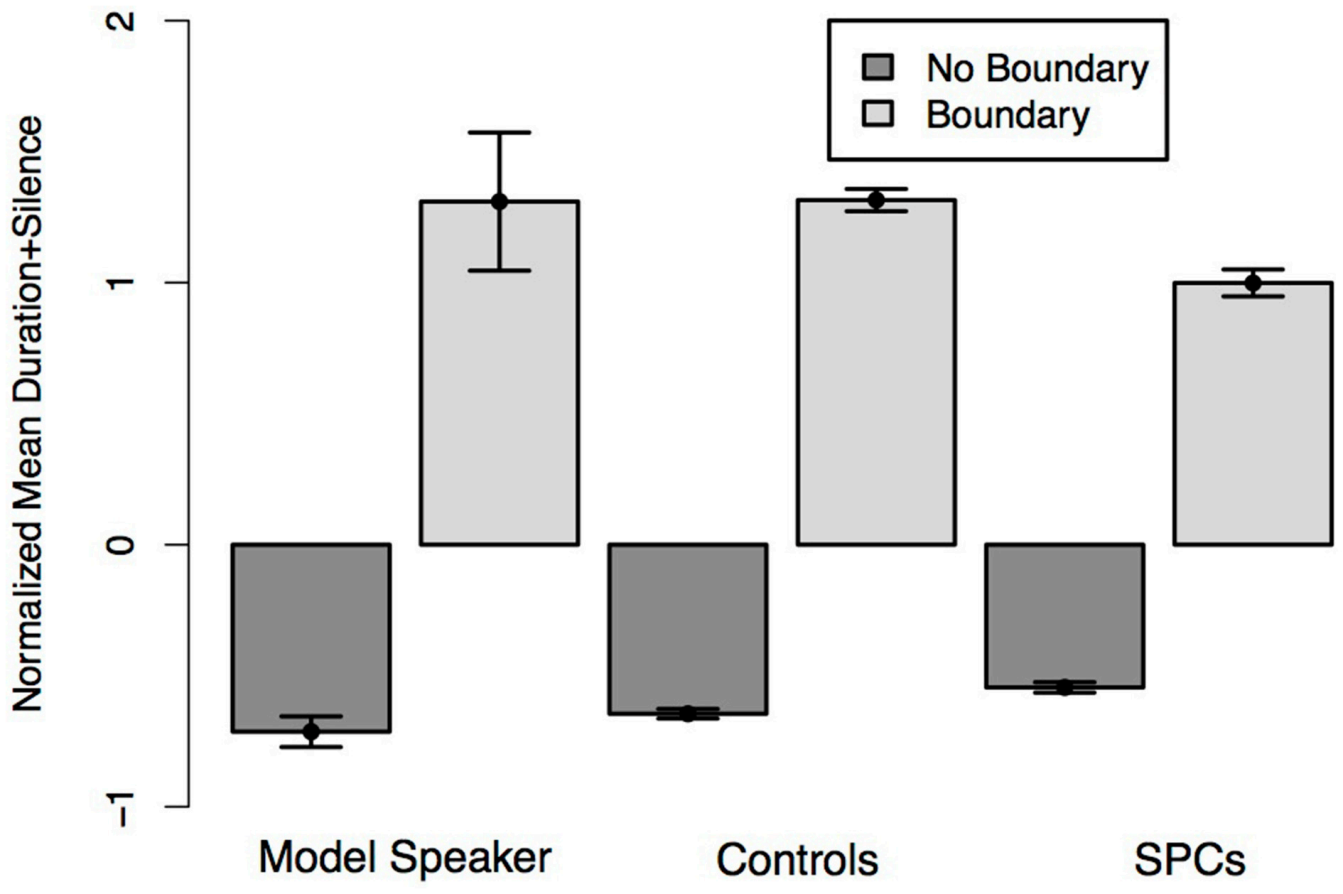
**Normalized average duration plus silence of words preceding hypothesized boundary locations and non-boundary locations in the Relative Clause sentences as produced by the Model Speaker, Controls, and SPCs**. Error bars represent standard errors.

We predicted that all speakers would produce the words that coincided with hypothesized boundary locations with longer durations than those that coincided with non-boundary locations. Moreover, we predicted that this effect would be moderated by Group such that Controls would realize a larger duration difference across the boundary conditions than SPCs. A mixed-effects linear regression predicting duration from boundary condition (boundary, no boundary) and Group revealed a main effect of boundary (*t* = −13.62), such that participants in both groups produced words preceding boundary locations with longer durations than those at non-boundary locations. There was a marginal effect of Group (*t* = −1.87), such that Controls produced all target words with longer relative durations. Critically, we observed a significant interaction between condition and group (*t* = 2.58) such that Controls once again produced greater durational differences between words occurring at boundaries and non-boundary locations (Figure 6).

### Relative clause: Pitch change

In order to determine whether Controls and SPCs differed in the extent to which they produced pitch cues to phrase boundaries, we compared the pitch variability of words that occurred at a hypothesized boundary location to those that did not. For each critical word in each production, including words that occurred at hypothesized boundary locations (e.g., room, chair) and hypothesized non-boundary locations (e.g., The, which, red), we computed a value of pitch change as the difference between the maximum and minimum F0 on that word and transformed this difference into semitones. Then, for each production, we averaged the pitch change values separately for the words adjacent to boundaries and the words non-adjacent to boundaries.

**Figure 7:**
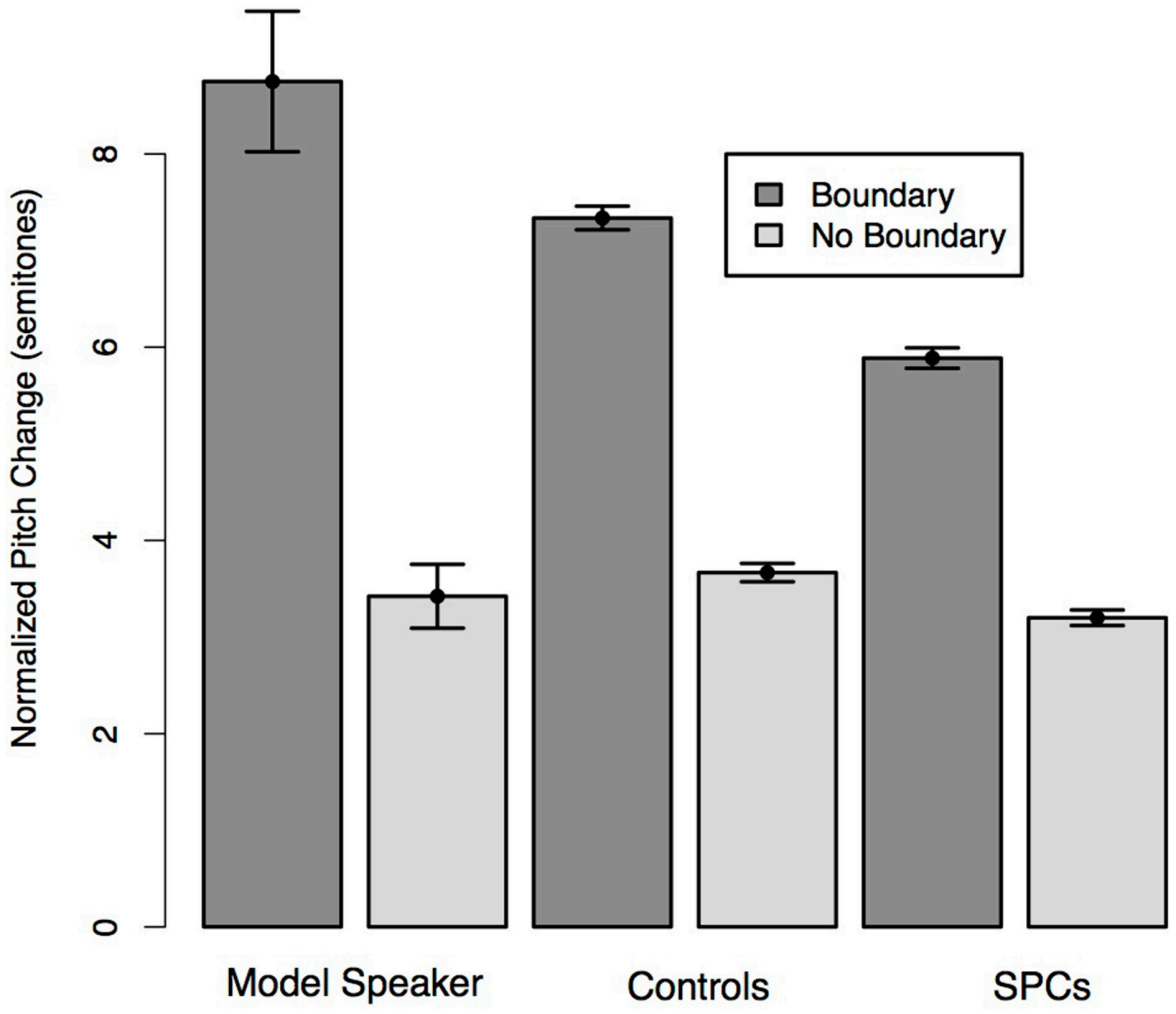
**Normalized pitch variation of words preceding hypothesized boundary locations and non-boundary locations in the Relative Clause sentences as produced by the Model Speaker, Controls, and SPCs**. Error bars represent standard errors.

We predicted that all speakers would produce the words that coincided with hypothesized boundary locations with greater pitch variation than those that coincided with non-boundary locations. Additionally, we predicted that this effect would be moderated by Group such that Controls would realize larger pitch variation across the boundary conditions than SPCs. A mixed-effects linear regression predicting pitch change from boundary condition (boundary, no boundary) and Group revealed a main effect of boundary (*t* = 18.55), such that participants in both groups produced words preceding boundary locations with greater pitch than those at non-boundary locations. We observed an effect of Group (*t* = 2.13), such that Controls produced all words with larger pitch variation than the SPCs. Moreover, we observed a significant interaction between condition and group (*t* = 2.87) such that Controls produced greater pitch differences between words occurring and boundaries and non-boundary locations than SPCs (Figure 7), thereby providing stronger relative cues to the location of a boundary than SPCs.

### Unambiguous coordinate structure: Duration

In order to determine whether Controls and SPCs differed in the extent to which they produced durational cues to phrase boundaries, we compared the duration of words that occurred at a hypothesized boundary location to those that did not. For each production from each speaker, we computed one value for the average normalized duration of words that occurred at a boundary location (e.g., dog, pen) and a second value for the average normalized duration of the first two words in each sentence, which occurred at predicted non-boundary locations (e.g., Ann, has).

**Figure 8:**
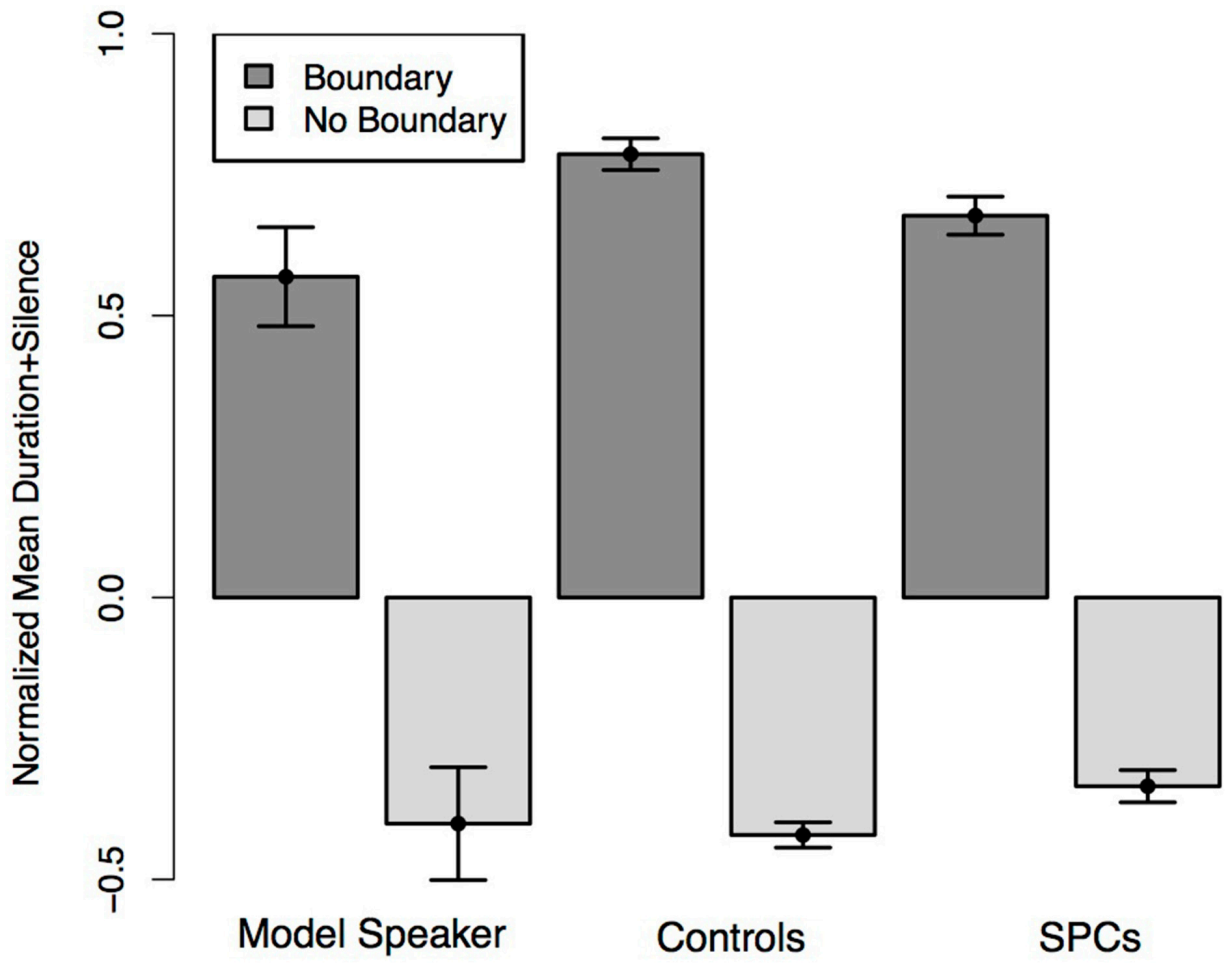
**Normalized average duration plus silence of words preceding hypothesized boundary locations and non-boundary locations in the Unambiguous Coordinate Structure sentences as produced by the Model Speaker, Controls, and SPCs**. Error bars represent standard errors.

We predicted that all speakers would produce the words that coincided with hypothesized boundary locations with longer durations than those that coincided with non-boundary locations. Furthermore, we predicted that this effect would be moderated by Group such that Controls would realize a larger duration difference across the boundary conditions than SPCs. A mixed-effects linear regression predicting duration from boundary condition (boundary, no boundary) and Group revealed a main effect of boundary (*t* = −13.46), such that participants in both groups produced words preceding boundary locations with longer durations than those at non-boundary locations. There was no main effect of group (*t* = 0.35) but we observed a significant interaction between condition and group (*t* = −3.31) such that Controls produced greater durational differences between words occurring at boundaries and non-boundary locations (Figure 8).

### Unambiguous coordinate structure: Pitch change

In order to determine whether Controls and SPCs differed in the extent to which they produced pitch cues to phrase boundaries, we compared the pitch variability of words that occurred at a hypothesized boundary location to those that did not. For each critical word in each production, including words that occurred at hypothesized boundary locations (e.g., dog, pen) and hypothesized non-boundary locations (e.g., Ann, has), we computed a value of pitch change as the difference between the maximum and minimum F0 on that word and transformed this value into semitones. Then, for each production, we averaged the pitch change values separately for the words adjacent to boundaries and the words non-adjacent to boundaries.

**Figure 9:**
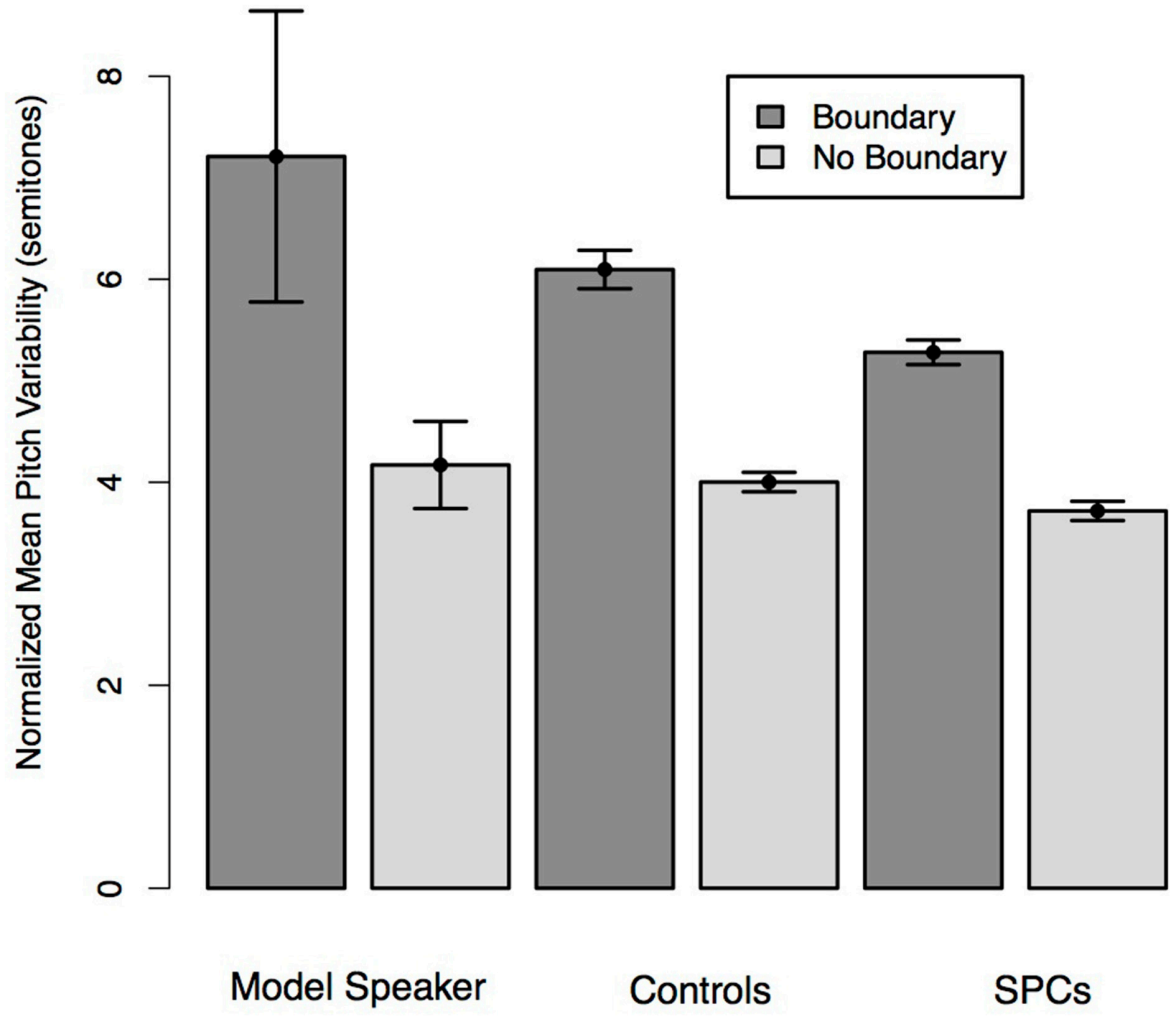
**Normalized pitch variation of words preceding hypothesized boundary locations and non-boundary locations in the Unambiguous Coordinate Structure sentences as produced by the Model Speaker, Controls, and SPCs**. Error bars represent standard errors.

We predicted that all speakers would produce the words that coincided with hypothesized boundary locations with longer durations than those that coincided with non-boundary locations. Moreover, we predicted that this effect would be moderated by Group such that Controls would realize a larger duration difference across the boundary conditions than SPCs. A mixed-effects linear regression predicting duration from boundary condition (boundary, no boundary) and Group revealed a main effect of boundary (*t* = −10.47), such that participants in both groups produced words preceding boundary locations with more pitch variability than those at non-boundary locations. We observed a marginal effect of Group (*t* = 1.96), such that Controls produced all words with higher relative pitch than the SPCs. Finally, we observed no interaction between Condition and Group (*t* = −1.52) although the differences were in the expected direction, such that Controls produced numerically larger pitch differences across conditions than SPCs (Figure 9).

## Discussion

The current study was designed to investigate the relationship between reading comprehension and imitated prosodic fluency in two groups of high school students who were matched on decoding ability. One group was comprised of students identified as SPCs, who demonstrated good decoding ability but relatively poor comprehension ability; the second (Control) group included individuals who demonstrated both good decoding ability and good comprehension ability. Participants in both groups imitated productions of a variety of sentence structures, including statements, questions, quotes, ambiguous coordinates, relative clauses, and unambiguous coordinates, which have been shown to reliably modulate prosodic phrasing and intonation. We assessed the two groups' prosody by comparing acoustic measures that have been shown to correspond to perceived phrasing and intonation; namely, pitch and duration.

Our predominant finding is that, across multiple constructions, SPCs consistently produced weaker duration cues to syntactic structure than Controls. Across all three phrasing constructions we tested (ambiguous coordinates, relative clauses, and unambiguous coordinates) Controls signaled boundaries with longer relative durations than SPCs. As described above, speakers cue syntactic phrase boundaries, which largely coincide with syntactic boundaries, with increased duration. This result suggests, therefore, that a particular source of difficulty for SPCs is the ability to assign (and perhaps recognize) linguistic constituent structure. We discuss this further below.

In addition to the larger duration cues produced by Controls compared to SPCs, we also observed some evidence that Controls realized larger pitch cues to syntactic and semantic structure. Specifically, in sentences containing relative clauses, although SPCs and Controls both produced words occurring at prosodic boundaries with significantly larger pitch variation than those occurring within prosodic phrases, Controls produced a significantly greater difference across conditions. Moreover, in two of the other three pitch features tested, namely, signaling boundaries in the unambiguous coordinate sentences, and signaling quotes in the Simple Quotative sentences, Controls produced numerically larger pitch variation than SPCs.

The lack of prominent pitch differences between SPCs and Controls is somewhat inconsistent with prior investigations of high schoolers' prosody. For example, Clay and Imlach (1971) and Dowhower (1991) demonstrated that good comprehenders produce larger pitch excursions at the ends of phrases than poor comprehenders. We believe that both methodological and group definitional choices may have reduced differences in pitch production in the current study. Specifically, our use of an imitation paradigm likely obscured differences between our groups, because participants were not required to generate a pitch contour themselves. Further, prior work has not considered comprehension and decoding ability independently and therefore some of these extant findings may have been driven by variation in decoding ability, given the need to read aloud. In addition, although the materials in the current study were based largely on Miller and Schwanenflugel (2006) (M&S), we did not replicate their findings. Again, this difference can likely be explained by differences between study populations and the production task: their study assessed the reading and production skill of 8–10-yr-olds who were not screened for word reading difficulties, while our participants ranged in age from 14 to 19 and had at least average word reading ability. Moreover, unlike M&S, who found that good readers made shorter pauses both within and between sentences, we found that Controls signaled larger prosodic boundaries. However, as M&S point out, the poor readers in their study made pauses that were inappropriately long, and “disrupted the flow of the sentence” (p. 851). We argue, therefore, that these results are likely due to difference in decoding skill between SPCs and Controls. Matching our good and poor comprehension groups on decoding skill, and providing then with a model production, meant that our participants produced sentences with more fluent prosody, which allowed us to assess more subtle relationships between the intended prosodic category and acoustic features.

It is likely the case that the poor readers in M&S's study were often disfluent (perhaps as a result of developing decoding skill), whereas our SPCs were fluent but did not produce acoustic cues to boundaries as effectively as Controls. In addition, while M&S reported that their participants generally produced basic quotatives with a flat contour, we observed large differences in pitch variation between the quote and the attributive phrase. Finally, M&S demonstrated that better readers realized larger F0 falls on declarative statements more effectively than poor readers. Once again, their description of the group's behavior suggests that the poor readers in M&S's study produced flat intonation while the better readers produced variable F0s. In contrast, participants in both groups in our study produced the same magnitude of expected contours for statements and questions. These differences demonstrate that prosodic fluency continues to develop in proficient readers, and that the relationship between fluency and comprehension also persists.

There are at least two possible explanations for the observed relationship between prosodic fluency and reading comprehension: The first possibility is that good reading comprehension leads to better prosodic fluency. We believe this interpretation is less likely given that children demonstrate adult-like prosody for many constructions well before they master reading (e.g., Wells et al., 2004). The second possibility is that prosodic fluency is one factor driving good comprehension such that readers who are more prosodically fluent also read with better comprehension. In what follows, we will argue that, in conjunction with findings from the adult prosody literature, the results of the current paper suggest that readers who effectively produce prosodic cues to syntactic structure when reading aloud similarly realize these cues when reading silently (Kuhn and Stahl, 2003). That is, readers who produce fluent explicit prosody also produce fluent implicit prosody.

The Implicit Prosody Hypothesis (IPH; Fodor, 2002) maintains that readers, when reading silently, activate prosodic representations of the text, similar to what they would produce if speaking aloud, and that these representations affect readers' interpretation of the text (see Breen, 2014, for a review). Evidence for the IPH comes from a variety of studies demonstrating that readers are sensitive to prosodic cues in reading in ways that are similar to listening. For example, overt prosodic boundaries can disambiguate syntactic structure by signaling to the listener that the upcoming material should not be attached to it. Indeed, there is considerable evidence from the psycholinguistic literature that listeners can use prosodic phrasing cues immediately to resolve syntactic ambiguity (e.g., Snedeker and Trueswell, 2003; Kraljic and Brennan, 2005). In addition, several studies of adult sentence processing have revealed evidence for implicit prosodic phrasing. For example, when reading silently, readers prefer phrase boundaries after long sentence constituents in the same way that they prefer to produce overt phrase boundaries when reading aloud (Hirose, 2003; Hwang and Steinhauer, 2011). In addition, Steinhauer and colleagues have demonstrated similar event-related potential (ERPs) signatures when participants are listening to sentences with overt phrase boundaries and when they are listening to sentences with implicit phrase boundaries (i.e., commas) (Steinhauer, 2003).

The IPH provides one way to account for our results. Specifically, if readers interpret written text with reference to their implicit prosodic representation, then we would expect readers who generate prosodic representations that don't reflect the syntactic structure of the text to have more difficulty comprehending what they read. Our results suggest that SPCs' specific comprehension deficit could be due, in part, to implicit prosodic representations that don't contain all of the relevant phrasing information and therefore don't facilitate syntactic parsing, which could lead to observed comprehension deficits.

In addition to evidence from the sentence processing literature that adult readers' implicit prosodic representations facilitate parsing, there is evidence that text-based cues to implicit prosody can also facilitate children's reading comprehension. For example, children exhibit higher fluency (characterized by fewer disfluencies) for phrases that are not interrupted by a line break (LeVasseur et al., 2006), and when text is visually grouped into phrases (LeVasseur et al., 2008). It may be the case that the visual cues to phrasing prime readers' implicit boundary representations which, as cues to syntactic structure, facilitate effective comprehension and production. Future work could explore whether text-based cues to syntactic structure would improve comprehension for SPCs.

The current study provides the first demonstration of differences in prosodic production between good and poor comprehenders who are matched on word decoding, furthering our understanding of what cognitive processes might be underlying comprehension differences between these groups. However, additional empirical work is required to clarify the current findings. First, although the imitation paradigm used in the current study allowed us for a focused investigation of prosodic cues in highly-controlled contexts, it also likely covered up some differences between the groups. Therefore, in a follow-up study we will assess prosodic differences between good and poor comprehenders in read (but not imitated) speech. Based on the current results, we expect that we will find similar differences between the groups such that good comprehenders will produce stronger acoustic cues to boundaries and prominence.

In addition to replicating the current findings with a different production paradigm, future studies will explore the extent to which SPCs and Controls differ in their *perception* of prosody. For example, our finding that SPCs produce weaker duration cues to prosodic boundaries suggests that they may also have difficulty perceiving durational cues to boundaries. For example, we predict that SPCs will have more difficulty recovering the correct interpretation of an ambiguous coordinate structure sentence, where the prosodic structure disambiguates the correct grouping of individuals, than Controls.

Differences in implicit prosodic skill between good and poor comprehenders are one possible explanation for our results, but this pattern of results could arise due to other differences between Controls and SPCs. For example, perhaps the SPCs are simply not as good at imitating the prosody of the model speaker, due to differences in executive functioning or working memory. Therefore, in future work, we will also assess whether working memory capacity contributes to differences in prosodic fluency between good and poor comprehenders.

The current results are significant for several reasons. First, to our knowledge, this is the first study of prosody in SPCs. This is a crucial methodological advance because, as evidenced by results from Miller and Schwanenflugel (2006), readers cannot effectively produce prosodic cues to syntactic and semantic structure if they are struggling to decode individual words. Secondly, this paper provides the first demonstration of individual differences in prosodic fluency using objective measures among high school students; prior work investigating the relationship between prosody and reading skill has focused almost exclusively on younger readers. Our results demonstrate that older readers' fluency continues to predict comprehension ability and suggest that secondary school readers could continue to benefit from targeted fluency training. Finally, the current study builds on a large psycholinguistic literature developed over the past 15 years looking specifically at the types of prosodic features young adult speakers employ to indicate the syntactic and semantic structure of sentences in ambiguous and non-ambiguous sentences. Understanding variability in how developing readers produce and comprehend these specific contours will inform our understanding both of reading processes, and of sentence processing development overall.

## Author contributions

MB: Data analysis and writing. LK: Data analysis and writing. JV: Stimulus and method development, writing. JK: Stimulus and method development. NL: Stimulus and method development, writing.

### Conflict of interest statement

The authors declare that the research was conducted in the absence of any commercial or financial relationships that could be construed as a potential conflict of interest.

## Footnotes

^1^Also Specific Comprehension Impairment (SCI).

^2^One participant in the SPC group was missing a value for IQ. We used the *mice* package in R to impute in IQ value for this participant using a linear prediction of WA and KTEA scores.

^3^We note that the significant effect of IQ in our sample appears to be driven primarily by higher than average IQ scores in our good comprehenders (viz., 7 out of our 16 control participants had IQ scores *above* the normal range).

^4^In fact, some of the sentences were globally ambiguous between a restrictive and non-restrictive reading, but the model speaker produced them all with phrase boundaries before the relative clause, thereby signaling a non-restrictive reading (Watson and Gibson, 2004).

^5^Psychology Software Tools and Inc. [E.-Prime, 2.0]. (2012). Available online at: http://www.pstnet.com

^6^We also fit models which, in addition to fixed effects of manipulation, group, and their interaction, also included fixed effects of Age and IQ (both centered). However, neither factor was significant in the final models, and their inclusion did not qualitatively change the results relative to the simpler models. Therefore, we report models without Age and IQ.
