# Supplementary material for: Imitated Prosodic Fluency Predicts Reading Comprehension Ability in Good and Poor High School Readers
Source: Front Psychol. 2016 Jul 19;7:1026. doi: 10.3389/fpsyg.2016.01026 (PMC4949254; doi:10.3389/fpsyg.2016.01026)
Supplement: Supplementary file 1 [file DataSheet1.docx]

# Appendix

**Statement**

1. Emily has painted a melon.

2. Anna will now play the piano.

3. Allison is starting ninth grade.

4. Stewart will go to college next year.

5. Alex is waiting at the bar.

6. Nicole finished making dinner.

7. Gwen gave a presentation on Monday.

8. Annie is buying an orange.

9. Dan is looking at an orange.

10. Ann is going to take the money.

**Yes-No Question**

1. Has Emily painted a melon?

2. Will Anna now play the piano?

3. Is Allison starting ninth grade?

4. Will Stewart go to college next year?

5. Is Alex waiting at the bar?

6. Has Nicole finished making dinner?

7. Did Gwen give a presentation on Monday?

8. Is Annie buying an orange?

9. Is Dan looking at an orange?

10. Is Ann going to take the money?

**Basic Quotatives**

1. "That sounds wonderful!” said Jane.

2. “I like it.” said Andrea.

3. “Good for you!” said Mark.

4. “It's not bad.” said Leslie.

5. “I want one!” Evan screamed.

6. “How awful!” Johnny said.

7. “It's for you.” Adam said.

8. “That's not good.” Peter replied.

9. “Let's play!” exclaimed Luther.

10. “That's new!” Rhonda said.

**Ambiguous Coordinate Structures**

*The 2-1 versions were presented with a comma after the second conjunct; the 2-2 versions were presented with a comma after the first conjunct.*

1. Ann(,) and Bobby(,) or Nancy, will come

2. Ann will teach Abe(,) and Bob(,) or Lenny

3. John will paint Al(,) and Dan(,) or Nina

4. George(,) and Donald(,) or Larry, will take art

5. Sal studies with Ally(,) and Nina(,) or Louie

6. Mathew carpools with Jim(,) and Zackary(,) or David

7. Dan(,) and Ryan(,) or Nicole, had dinner

8. Ann(,) and Doug(,) or Lee, will take you home

9. Dan(,) and Lenny(,) or Edward, will leave

10. Ann(,) and Roy(,) or Dan, will leave London

**Relative Clauses**

1. Their father, who was in the army, stood in the yard.

2. After the rain, which ended at nine, we walked around the pond.

3. Laurie's grandma, who is a lawyer, did not like the brochure.

4. The girl, who was injured, ran from the tornado.

5. The Dude, who was on a mission, broke into the house.

6. The driveway, which was muddy, led to the mansion.

7. The novel, which she finished, sat on the nightstand.

8. The star player, who joined the team, was a receiver.

9. The widow, who was devastated, received many flowers.

10. The journey, which I took alone, felt like it would never end.

11. The groom, who was on edge, drank too much.

12. The chaperones, who volunteered, forgot to come.

13. The apple, which she bought at the farm, was fresh.

14. The room, which had a red chair, caught Mandy's eye.

15. His wagon, which only had three wheels, was noisy.

16. The play, which Bobby wrote junior year, was about zombies.

**Unambiguous Coordinate Structures**

1. Ann has a dog, a pen, and a mug.

2. Ann bought a rose, a bird, and jelly.

3. John wants a bagel, butter, and jam.

4. George loves history, math, and English class.

5. Alex takes a car, a taxi, or a train to work.

6. Nicole made chicken, bread, and pasta.

7. Ben talked about the baby, the doll, and the lion.

8. Ann bought a pie, a bag, and a lime.

9. Dan plays with Rob, Nathan, and Morgan.

10. Ann looked at Ed, Mary, and Nellie.
